# Biologic Therapies in the Management of Sports-Related Tendon and Ligament Injuries: A Narrative Review

**DOI:** 10.7759/cureus.84556

**Published:** 2025-05-21

**Authors:** David Koshy, Daniel I Koshy, Emma Ooi

**Affiliations:** 1 Orthopaedics, University Hospitals Plymouth, Plymouth, GBR; 2 Orthopaedics, St John of God Midland Public Hospital, Perth, AUS; 3 Trauma and Orthopaedics, Royal London Hospital, London, GBR; 4 Internal Medicine, Queen Elizabeth Hospital Birmingham, Birmingham, GBR

**Keywords:** biologic therapy, mesenchymal stem cells (mscs), orthopaedic sports medicine, platelet-rich plasma, regenerative medicine, soft tissue injury, sports medicine

## Abstract

Sports-related tendon and ligament injuries are common among athletes and active individuals, often resulting in prolonged recovery and compromised performance. Traditional management strategies, including physiotherapy and surgical repair, may not yield optimal outcomes, prompting growing interest in biologic therapies such as platelet-rich plasma (PRP), mesenchymal stem cells (MSCs), and other regenerative modalities. This targeted narrative review synthesizes current evidence on the application of biologic therapies in treating tendon and ligament injuries, emphasizing their mechanisms of action, clinical efficacy, and limitations. Relevant studies were identified through a focused search of PubMed and Google Scholar from 2010 to 2024. PRP demonstrates sustained pain and function improvement in chronic tendinopathies, whereas MSCs show promise in enhancing graft integrity in ligament reconstruction; however, variability in preparation protocols and limited long-term randomized controlled trials constrain firm conclusions. Emerging approaches, including exosomes and scaffold-based delivery systems, offer new avenues for enhancing tissue healing. Biologic interventions hold considerable promise in augmenting soft tissue injury treatment, but standardized protocols and further high-quality trials are needed to support widespread adoption.

## Introduction and background

Musculoskeletal injuries are a leading cause of morbidity in athletes and active individuals, with tendinous and ligamentous injuries representing a substantial proportion of cases. Due to their limited vascularity and complex bio-mechanical roles, these structures often demonstrate poor intrinsic healing capacity and prolonged recovery periods [1]. For elite athletes, this can result in extended time away from competition and an increased risk of re-injury or incomplete functional recovery.

Conventional management approaches, including rest, physical therapy, non-steroidal anti-inflammatory drugs (NSAIDs), and surgical reconstruction, form the mainstay of treatment. However, these methods are frequently associated with suboptimal tissue regeneration and variable clinical outcomes [2]. In response to these limitations, biologic therapies have emerged as a promising adjunct or alternative, aiming to enhance endogenous repair mechanisms and improve structural and functional outcomes. Biologic therapies, such as platelet-rich plasma (PRP), autologous conditioned serum (ACS), mesenchymal stem cells (MSCs), and other cell-based products, are designed to deliver bioactive molecules and regenerative cells directly to the injury site. These modalities are hypothesized to modulate inflammation, promote angiogenesis, and facilitate matrix remodelling, potentially accelerating the healing process and improving tissue quality [3].

Despite growing interest and widespread clinical use, the evidence base for biologic therapies remains heterogeneous and, in some areas, inconclusive. Questions persist regarding optimal formulations, delivery protocols, patient selection criteria, and long-term safety and efficacy. Furthermore, regulatory challenges and the lack of standardized treatment guidelines hinder broad clinical adoption.

This narrative review aims to critically examine the current role of biologic therapies in managing sports-related injuries, with a particular focus on ligament and tendon pathologies. It synthesizes the available evidence on efficacy, highlights key limitations in the current literature, and explores emerging trends and future directions in regenerative sports medicine. This narrative review is based on a targeted literature search conducted in PubMed and Google Scholar (2010-2024), using terms such as "platelet-rich plasma", "stem cells", "biologics", "tendinopathy", and "ligament injury". Relevant clinical trials, meta-analyses, and high-impact reviews were prioritized.

## Review

Overview of biologic therapies

Biologic therapies in sports medicine encompass a diverse range of interventions that aim to modulate the healing environment through cellular and molecular mechanisms. These therapies are designed to enhance tissue regeneration, reduce inflammation, and support functional recovery in musculoskeletal injuries. The most widely used biologic agents include PRP, MSCs, ACS, and other emerging modalities such as exosomes and amniotic tissue derivatives.

Platelet-Rich Plasma

PRP is an autologous blood derivative that concentrates platelets above baseline levels and delivers a rich milieu of growth factors, including platelet-derived growth factor (PDGF), vascular endothelial growth factor (VEGF), and transforming growth factor-beta (TGF-β), which are implicated in tissue repair and angiogenesis [3,4]. PRP can be classified into leukocyte-rich and leukocyte-poor formulations, with differing effects on inflammation and healing depending on the clinical context [5].

Despite its widespread use, there remains considerable heterogeneity in PRP preparation methods, platelet concentrations, and injection protocols, contributing to variability in clinical outcomes [6]. Nevertheless, PRP has shown promise in managing tendinopathies, muscle injuries, and ligamentous lesions, particularly in chronic, degenerative settings.

Mesenchymal Stem Cells

MSCs are multipotent stromal cells capable of differentiating into tenocytes, chondrocytes, and other musculoskeletal lineages. They exert their therapeutic effects not only through differentiation but also via paracrine signalling, modulating inflammation, and promoting extracellular matrix remodelling [7]. MSCs can be harvested from bone marrow, adipose tissue, umbilical cord, and other sources, each with distinct cellular yields and immunomodulatory profiles.

In preclinical models, MSCs have demonstrated efficacy in enhancing tendon-to-bone healing and improving structural integrity of ligaments post-injury [8]. Early-phase clinical trials in humans suggest potential benefits in rotator cuff repair and anterior cruciate ligament (ACL) reconstruction, although large-scale randomized controlled trials (RCTs) are still needed to validate these findings [9].

Autologous Conditioned Serum

ACS, also known by its commercial name Orthokine, is derived from autologous blood incubated with glass beads to stimulate monocyte production of anti-inflammatory cytokines such as interleukin-1 receptor antagonist (IL-1Ra) [10]. Initially developed for osteoarthritis, ACS has also been explored in the treatment of soft tissue injuries due to its potential to modulate catabolic pathways associated with chronic inflammation.

Evidence supporting ACS in tendon or ligament injuries is currently limited to small-scale studies, but it remains an area of active investigation, particularly in elite athletic populations.

Amniotic and Placental Tissue Derivatives

Amniotic membrane and placental-derived products contain extracellular matrix proteins, growth factors, and cytokines that may support tissue regeneration and modulate immune responses. These allografts are typically processed into injectable or implantable forms and have gained traction for their potential use in chronic tendinopathies and surgical augmentation [11].

Though preclinical data are promising, clinical studies remain scarce, and standardized processing methods are lacking. Furthermore, regulatory classification of these products varies by country, affecting their clinical accessibility.

Emerging Therapies: Exosomes and Gene Therapy

Exosomes, nanoscale extracellular vesicles released by cells such as MSCs, represent a novel frontier in regenerative medicine. They carry proteins, lipids, and nucleic acids that can influence cell behaviour, enhance angiogenesis, and regulate inflammation without the immunologic risks of whole-cell transplantation [12]. Similarly, gene therapy approaches aim to deliver genes encoding regenerative cytokines or growth factors directly to injured tissue, though clinical application remains experimental.

Applications in tendon injuries

Tendinopathies are among the most common musculoskeletal disorders encountered in both athletes and the general population. They include a range of acute and chronic injuries, such as rotator cuff tendinopathy, Achilles tendinopathy, and lateral epicondylitis, often resulting from overuse and mechanical overload. These conditions are notoriously difficult to treat due to poor vascularity, a low cellular turnover rate, and a propensity for chronic degeneration rather than acute inflammation [13,14]. In this context, biologic therapies have emerged as potential tools to promote tendon regeneration and accelerate return to function.

Platelet-Rich Plasma in Tendinopathies

PRP has been the most extensively studied biologic intervention for tendon injuries. Its rich content of growth factors, including PDGF, VEGF, and TGF-β, is thought to promote tenocyte proliferation, collagen synthesis, and angiogenesis [3,4].

In lateral epicondylitis (tennis elbow), multiple RCTs have demonstrated significant improvements in pain and function compared to corticosteroids and placebo, with benefits sustained over longer follow-up periods [3,15]. For Achilles tendinopathy, the evidence is more equivocal. While some studies suggest improved tendon structure and reduced pain scores [16], others show no significant difference compared to saline injections or eccentric loading programs [17].

The variability in outcomes is attributed in part to heterogeneity in PRP formulations (leukocyte-rich vs. poor), injection protocols, and injury chronicity [18]. Meta-analyses suggest that PRP may be most effective in chronic, degenerative tendinopathies rather than acute tears.

Mesenchymal Stem Cells

MSCs offer a more potent regenerative option due to their dual role in differentiating into tenocytes and secreting paracrine mediators that support matrix remodelling and reduce inflammation. Preclinical studies in tendon injury models have shown improved collagen organization, increased mechanical strength, and accelerated healing following MSC application [19].

Clinically, MSCs have been studied primarily in the context of rotator cuff pathology. A landmark study by Hernigou et al. (2014) found that patients receiving bone marrow-derived MSCs at the time of rotator cuff repair had a significantly lower retear rate and superior structural integrity at 10-year follow-up compared to controls [20].

Amniotic and Placental Tissue Derivatives

Amniotic tissue products, rich in cytokines, extracellular matrix proteins, and hyaluronic acid, have gained popularity as injectable or implantable biologics for chronic tendon injuries. These derivatives have demonstrated anti-inflammatory, anti-fibrotic, and pro-regenerative properties in preclinical models [21].

Other Biologic Approaches

ACS, though traditionally used in osteoarthritis, is being explored in chronic tendinopathies due to its IL-1Ra content, which may modulate chronic inflammation seen in degenerative tendons [10]. However, its clinical evidence base in tendon injuries remains sparse.

Exosomes, derived from MSCs or other cell types, represent a novel, cell-free approach. In preclinical studies, exosomes have been shown to enhance tendon healing through upregulation of type I collagen and downregulation of pro-inflammatory cytokines [22]. Clinical translation is still in the early stages.

Applications in ligament injuries

Ligament injuries, particularly of the ACL, are highly prevalent in both recreational and professional athletes. These injuries pose significant clinical challenges due to their limited healing potential and high risk of long-term sequelae such as joint instability and early-onset osteoarthritis [23]. Surgical reconstruction remains the standard of care for complete ligament tears, especially in high-demand individuals. However, biologic therapies are being increasingly explored both as adjuncts to surgical repair and as stand-alone treatments in partial tears, with the aim of enhancing graft incorporation, promoting native tissue healing, and reducing reinjury rates.

Platelet-Rich Plasma in Ligament Healing

PRP has been used in ACL injuries to augment graft healing by stimulating angiogenesis, collagen synthesis, and cellular proliferation at the graft-bone interface [24]. In vitro and animal studies have consistently shown improved biomechanical strength and histological quality of the healing ligament with PRP application [25,26].

Clinically, the data are mixed. Some RCTs have demonstrated that PRP can accelerate graft maturation, as assessed by MRI signal intensity, and reduce postoperative effusion [27]. However, most studies fail to show significant improvements in long-term functional outcomes, ligament laxity, or graft failure rates [28]. PRP may offer more consistent benefits in the early phases of recovery, but appears insufficient as a stand-alone therapy for complete ligament injuries.

Mesenchymal Stem Cells

MSCs offer promise in ligament healing through both direct differentiation into ligament fibroblasts and by releasing trophic factors that enhance vascularization and matrix remodelling [7]. Preclinical studies have demonstrated that MSCs, especially when delivered in a scaffold or hydrogel, can significantly improve the biomechanical properties of healing ligaments [29].

Clinical evidence, though still limited, is encouraging. In a prospective cohort study by Centeno et al. (2015), intra-articular injection of bone marrow-derived MSCs for partial ACL tears showed improvements in knee stability and function, with MRI evidence of partial tissue regeneration [30]. However, randomized trials remain scarce, and optimal delivery methods and cell dosages are yet to be established.

Biologic Scaffolds and Tissue Engineering Approaches

Biologic scaffolds composed of collagen, hyaluronic acid, or silk-based materials are being explored as vehicles to deliver PRP, stem cells, or growth factors directly to the injury site. One of the most prominent examples is the bridge-enhanced ACL repair (BEAR) technique, which uses a bioactive scaffold saturated with autologous blood to bridge the torn ACL ends. In a multicentre RCT, the BEAR technique was shown to be non-inferior to traditional ACL reconstruction in terms of knee stability and function at two years, with a lower rate of osteoarthritis progression [31].

Such approaches mark a shift toward biologically augmented repair rather than reconstruction, potentially preserving native tissue and proprioception. However, BEAR and similar technologies are currently limited to specific patient populations (e.g., skeletally mature patients with mid-substance ACL tears) and are not yet widely available.

Other Biologic Modalities

ACS and amniotic tissue derivatives have not been widely studied in ligament healing but show potential based on their anti-inflammatory profiles. Exosomes, a cutting-edge cell-free therapy, are being explored for their ability to modulate the post-injury inflammatory response and enhance collagen organization. In preclinical ACL models, exosome-treated grafts demonstrated superior tensile strength and histological organization [32]. While still early in development, exosome-based therapies may offer a scalable and less immunogenic alternative to whole-cell approaches.

Challenges and controversies

While biologic therapies hold significant promise for enhancing the healing of tendon and ligament injuries, their integration into mainstream sports medicine has been hindered by numerous scientific, clinical, and regulatory challenges. Key areas of contention include variability in treatment protocols, inconsistent clinical outcomes, limited regulatory oversight, and a lack of high-quality evidence to support widespread adoption.

Lack of Standardization

A central limitation across studies is the heterogeneity in biological preparation and application protocols. For example, PRP can vary widely in platelet concentration, leukocyte content (leukocyte-rich vs. leukocyte-poor), activation methods, and injection frequency [33,34]. These variations contribute to inconsistent results across trials and complicate meta-analyses, making it difficult to draw definitive conclusions regarding efficacy.

Similarly, MSC therapies differ in terms of cell source (e.g., bone marrow, adipose tissue, and umbilical cord), harvesting techniques, expansion protocols, and delivery vehicles. Without standardized processing and dosing parameters, comparing outcomes between studies remains problematic.

Inconsistent Clinical Outcomes

Although preclinical models consistently show promising results for PRP, MSCs, and other biologics, translation to human trials has been inconsistent. Some clinical studies report significant improvements in pain, function, and tissue integrity, while others demonstrate no benefit over placebo or standard care [14]. In many cases, the heterogeneity in patient populations, injury chronicity, and concomitant therapies further clouds interpretation.

Moreover, the placebo effect in biologic injections is substantial, particularly in the context of musculoskeletal pain. Studies have shown that saline or sham injections can produce meaningful clinical improvements, underscoring the importance of properly blinded, placebo-controlled designs [35].

Regulatory and Ethical Concerns

Regulation of biologic therapies remains inconsistent across jurisdictions. In the United States, the FDA regulates cell and tissue-based products under the Human Cells, Tissues, and Cellular and Tissue-Based Products (HCT/P) framework. However, ambiguity exists regarding what constitutes "minimal manipulation" or "homologous use", leading to widespread off-label applications of biologics, especially in the private sector [36].

Ethical concerns also arise around the commercialization of biologics without robust evidence of efficacy. Some clinics market unproven stem cell therapies at high costs, targeting vulnerable populations with misleading claims [37]. These practices risk undermining public trust and may impede legitimate scientific progress.

Cost and Accessibility

Biologic therapies are often expensive, with costs for MSC procedures ranging from several thousand to tens of thousands of dollars, often not covered by insurance. This raises questions about cost-effectiveness, particularly when benefits over conventional therapies are marginal or uncertain. Additionally, the need for specialized equipment and expertise limits the accessibility of biologics to high-resource settings or elite sports organizations, creating disparities in care.

Long-Term Safety and Efficacy

Long-term data on biologic therapies remain scarce. While short-term studies generally show favourable safety profiles, the potential risks of immunogenicity, infection, ectopic tissue formation, or tumorigenesis, particularly with MSCs, require continued surveillance [38]. Moreover, the durability of clinical benefit beyond one to two years is seldom reported, making it difficult to assess true long-term efficacy.

Future directions

As regenerative medicine continues to evolve, the role of biologic therapies in managing sports-related tendon and ligament injuries is poised to expand. While current applications are promising yet inconsistent, future advancements in biologic optimization, delivery systems, patient stratification, and regulatory frameworks may help unlock the full potential of these therapies.

Standardization of Biologic Products

One of the foremost priorities in biologic therapy research is the development of standardized protocols for preparation, characterization, and administration. For PRP, this includes consistent classification based on platelet concentration, leukocyte content, activation status, and volume delivered [34,39]. Similarly, for MSCs, standardized reporting of cell source, isolation technique, viability, and surface markers is essential to ensure reproducibility and clinical comparability.

International consensus guidelines and registries, such as the MIBO (Minimum Information for Studies Evaluating Biologics in Orthopaedics) initiative, are emerging to promote methodological transparency and facilitate meta-analyses [40].

Precision Medicine and Patient Selection

Advances in molecular diagnostics and biomarker research may enable personalized biologic interventions tailored to specific injury types, stages, and patient profiles. For instance, emerging evidence suggests that patients with chronic degenerative tendinopathies may respond more favourably to PRP than those with acute inflammatory lesions [18]. Identifying predictive biomarkers for biologic responsiveness could guide clinical decision-making and optimize outcomes.

Novel Delivery Platforms and Scaffolds

Next-generation delivery platforms are being developed to enhance the localization, retention, and activity of biologic agents. These include hydrogel matrices, collagen scaffolds, nanocarriers, and bioactive sutures that allow for sustained release of growth factors or stem cells at the injury site.

Techniques such as ultrasound-guided injections and arthroscopic delivery systems are improving the precision of biologic administration, potentially enhancing efficacy and reducing adverse effects. Scaffold-assisted techniques like BEAR exemplify the shift toward biologically augmented tissue repair rather than reconstruction [41].

Exosome and Gene-Based Therapies

Cell-free therapies, particularly extracellular vesicles such as exosomes, are gaining traction as alternatives to cell-based treatments. Exosomes derived from MSCs have been shown to promote tendon and ligament regeneration via paracrine mechanisms, offering advantages such as lower immunogenicity, easier storage, and reduced regulatory hurdles.

Gene therapy, though still largely experimental, holds potential for delivering anabolic or anti-inflammatory factors directly to injured tissues. Viral and non-viral vectors are being investigated for the local delivery of genes encoding VEGF, bone morphogenetic protein (BMP), and insulin-like growth factor-1 (IGF-1), aiming to enhance the regenerative microenvironment [42].

Long-Term Studies and Health Economic Analyses

There is a pressing need for long-term, high-quality RCTs with robust outcome measures to assess the durability, safety, and comparative effectiveness of biologic therapies. These trials should incorporate both objective structural metrics (e.g., MRI, histology, and biomechanical testing) and validated patient-reported outcomes.

Additionally, economic evaluations are essential to determine the cost-effectiveness of biologics compared to standard treatments. This is particularly important as the high cost of certain biologic interventions may limit their accessibility and reimbursement [43].

## Conclusions

Biologic therapies such as PRP, MSCs, and emerging cell-free approaches offer a promising adjunct to conventional treatments for tendon and ligament injuries in sports medicine. While preclinical studies and early clinical data suggest benefits in enhancing tissue repair and reducing recovery time, clinical outcomes remain variable due to heterogeneity in preparation methods, patient selection, and delivery protocols. Standardization, rigorous randomized trials, and long-term follow-up are urgently needed to clarify their efficacy, safety, and cost-effectiveness. With continued research and technological refinement, biologics have the potential to transform the management of musculoskeletal injuries and support optimal recovery in both athletic and general populations.
